# Synthesis of coimmobilized microorganisms for the removal of cadmium from cadmium‐contaminated rice flour

**DOI:** 10.1002/fsn3.2427

**Published:** 2021-06-25

**Authors:** Fang Zhao, Hu Zhang, Pianpian Yan, Yuwei Chen, Qian Wu, Min Fang, Yongning Wu, Zhiyong Gong

**Affiliations:** ^1^ Key Laboratory for Deep Processing of Major Grain and Oil of Ministry of Education Wuhan Polytechnic University Wuhan China; ^2^ NHC Key Laboratory of Food Safety Risk Assessment Food Safety Research Unit (2019RU014) of Chinese Academy of Medical Science China National Center for Food Safety Risk Assessment Beijing China

**Keywords:** cadmium, co‐immobilized, fermentation, *L*. *plantarum*

## Abstract

China has the greatest rice production in the world, but the problem of heavy metal pollution in rice is becoming increasingly serious. The present study examined a microbial immobilization method to remove cadmium (Cd) in rice flour. The study demonstrated that *Lactobacillus* *plantarum* (*L*. *plantarum*) exhibited the best removal effect, but the microorganisms were difficult to separate from rice flour. Diatomaceous earth coimmobilized microbial pellets (DECIMPs) were prepared using coimmobilized *L*. *plantarum* with sodium alginate (SA, 3%), polyvinyl alcohol (PVA, 2%), and diatomaceous earth (DE, 1%). Compared with microbial fermentation, the immobilized pellets had less influence on rice quality, and Cd removal rates of sample 1 (0.459 ± 0.006 mg/kg) and 2 (0.873 ± 0.031 mg/kg) reached 90.01% ± 1.01% (0.051 ± 0.003 mg/kg) and 91.80% ± 0.54% (0.068 ± 0.034 mg/kg), which were significantly higher than free microbial fermentation. In addition, microbial was easily separated. These results show that DECIMPs fermentation is an effective means of removing Cd from rice and could be considered as a strategy for the development of Cd‐free rice‐based foods.

## Graphical abstract

*Lactobacillus* *plantarum* exhibited the best cadmium removal effect among the seven strains. Higher cadmium removal rate than free microbe. Immobilized microorganisms have little effect on rice nutritional quality.

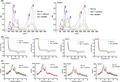

## 1 INTRODUCTION

Cadmium (Cd) pollution, especially in crops, is becoming increasingly serious due to the misuse of Cd in human activities (Ettler et al., 2012). In China, nearly 7% of the soil and 1.3 × 10^7^ hm^2^ acres of agricultural land are contaminated with Cd. On average, 12 million tons of rice are contaminated with Cd annually in China (Jiguang et al., 2003; Sun et al., 2016). Due to the accumulation of Cd in the soil, Cd absorption in crops which inevitably leads to Cd entering the human body through the food chain (Rahimzadeh et al., 2017). The residue of Cd in the human body is 3%–5%, but it exhibits a half‐life of up to 10–30 years in the liver and kidney (Kjellström & Nordberg, 1978). Toxicological studies demonstrated that long‐term exposure to dietary Cd led to kidney and bone damage (Chen et al., 2006).

Rice is a staple cereal crop in Asia and is the main cause of Cd exposure in Chinese, and people who live in the south are twice as likely to be exposed as those who live in the north (Song et al., 2017). The limit of Cd in rice is less than 0.200 mg/kg in China depending on Chinese food safety standard GB2762 (Lu & Toy, 2009). Our previous research showed that the excess rate of Cd exposure in Chinese rice is over 10%. Using the simple approach, such as burning and landfills, could lead to material resource waste and great environmental pollution, which further aggravates the tight Chinese grain supply. The safe and rational use of rice with excessive Cd is an ongoing question.

Traditional methods, such as acid soak, water soak, and heat treatment, can removing Cd from rice (Li et al., 2015; Mihucz et al., 2010). These methods have some disadvantages, such as low efficiency and impaired nutrition. Microbial fermentation to reduce heavy metals has been an active area of research in the field of food and environment science (Volesky, 2001; Wu et al., 2016; Zhang et al., 2018), but microorganisms are difficult to isolate and the survival rate of microorganisms is also a key factor (Mrozik & Piotrowska‐Seget, 2010). Immobilization technology, which immobilizes microorganisms on a medium, has become a hot technology (Cassidy et al., 1997; Karel et al., 1985). Cai et al. (2016) used SA and PVA as carriers to prepare immobilized *penicillium* *janthinillum* via embedding, which had good adsorption for Cu, Pb, and Cd in water and could be recycled. Immobilized materials have better adsorption performance than free microorganisms due to their high biomass, cell reuse, high mechanical strength, and high resistance to toxic chemicals (Kadimpati et al., 2013).

The present study used diatomite as an immobilized carrier to immobilize microorganisms for the removal of Cd in rice flour. The preparation technology and application conditions of immobilized microorganism pellets were optimized, and the quality of rice flour after Cd removal was determined. Microbes cannot be separated from food, which is a shortcoming, but the present study represents the first application of immobilized microorganisms for Cd removal in rice flour.

## 2 MATERIALS AND METHODS

### 2.1 Rice samples and chemicals

Cadmium‐contaminated rice samples 1 (0.459 ± 0.006 mg/kg) and 2 (0.873 ± 0.031 mg/kg) were collected from Hunan Province. The rice was dried, hulled, milled, and analyzed using graphite furnace atomic absorption spectrophotometry (GFAAS, PerkinElmer) in Wuhan Polytechnic University. A Cd standard solution was purchased from the National Center of Analysis and Testing for Nonferrous Metals and Electronic Materials. HNO_3_ (≥99.8%), KBr (≥99.8%), sodium alginate (SA, ≥99.5%), polyvinyl alcohol (PVA, ≥99.5%), CaCl_2_ (≥99.5%), diatomaceous earth (DE, ≥99.5), and Cd chloride (CdCl_2_, ≥99.5%) were obtained from Sinopharm Chemical Reagent Co., Ltd, de Man, Rogosa, Sharpe (MRS) broth and Malt Extract Agar were purchased from Qingdao Hope Bio‐Technology Co., Ltd. The total starch detection kit was obtained from Megazyme. A brown rice flour quality control sample was purchased from the Academy of State Administration of Grain.

### 2.2 Bacterial strains and culture

*Bifidobacterium* *longum* (BL, CCTCC AB 2010209), *Lactobacillus* *reuteri* (*L*. *reuteri*, CCTCC AB 2014289), and *Lactobacillus* *plantarum* (*L*. *plantarum*, CCTCC AB 2010210) were purchased from the China Center for Type Culture Collection (CCTCC) in Wuhan University. *Streptococcus* *thermophiles* (ST, 1.1864) and *saccharomyces* *cerevisiae* (SC, 2.3866) were obtained from the China General Microbiological Culture Collection Center (CGMCC). *Angel* *yeast* and *five* *bacterial* *leavening* *agents* (FBLA) were purchased from local markets. SC and Angel yeast were cultured in Malt Extract Agar at 25°C for 18 hr. The BL, *L*. *plantarum*, *L*. *reuteri*, ST, and FBLA were cultured in de Man, Rogosa, Sharpe (MRS) broth at 37°C for 18 hr.

### 2.3 Determination of Cd removal rate

Accurately weighed 0.2–0.3 g sample in a polytetrafluoroethylene (PTFE) vessel and 7 ml HNO_3_ was added to the PTFE digestion tubes. The tubes were incubated for 2 hr to digestion and placed in a microwave digestion system (Anton). Then, put it in an acid‐driven processor (Shanghai Broadcom Chemical Technology Co., Ltd.) to remove the superabundance of nitric acid. After it cooled, the solution was transferred to 50‐ml plastic centrifuge tubes and diluted with nitric acid (0.2%)–25 ml.

The Cd concentration was determined using GFAAS. The wavelength was 228.8 nm, the slit was 0.7 nm, and the lamp current was 4 mA. The following formula was used to calculate Cd removal rate:

Removal (%) = (C_0_–C)/C_0_ × 100% (1)

where C_0_ and C were the concentrations of Cd in rice flour before and after fermentation.

### 2.4 Microbial fermentation for Cd removal

#### 2.4.1 Optimization of the fermentation strains

Accurately weighted 50.0 g rice, added the microorganism, which was soaked in ultrapure water. The conditions of microbial fermentation time (24 hr), inoculum concentration (3%), and ratio of solid‐liquid (1:3) were selected to examine the optimum strain. The fermentation temperature was the optimum temperature of each strain. After fermentation, the fermentation broth was removed and washed three times with Milli‐Q water. Drying and grinding at 40°C for the measurements. As a control, rice was soaked in sterile water without fermentation strains.

#### 2.4.2 Optimization of the fermentation procedure

A single‐factor experiment was used to determine the optimal conditions for removing the Cd. The effects of different inoculum concentrations (1%, 2%, 3%, 4%, and 5%), solid‐liquid ratios (1:1, 1:2, 1:3, 1:4, and 1:5), and fermentation times (12, 24, 36, 48, and 60 hr) on Cd removal from rice were investigated as previously described (Zhai et al., 2019; Zhang et al., 2017). The remaining conditions were performed according to the fermentation method with the above‐mentioned procedures. An orthogonal test was designed to identify the optimal conditions and the order of the primary and secondary factors for Cd removal.

### 2.5 Diatomaceous earth coimmobilized microbial pellets fermentation for Cd removal

#### 2.5.1 Preparation of DECIMPs

Approximately 1.5 g PVA and SA were dissolved in Milli‐Q water (45 ml) at room temperature. The mixture was heated to dissolve the materials and sterilized at 120°C. 5 ml of *L*. *plantarum* suspension (OD_620_ = 1) and DE (0.5 g) were added after cooling, and the solution was mixed. The mixture was added slowly to cross‐link with CaCl_2_ at 4°C for 24 hr. Pellets were formed and rinsed three times with ultrapure water. Microbial immobilized pellets (MIPs), diatomite immobilized pellets (DIPs), and empty pellets (Eps) were prepared according to the above‐mentioned method.

#### 2.5.2 Optimization of the preparation process for DECIMPs

Accurately weighted 50.0 g rice flour, the adsorption capacity of Cd was taken as the index, single‐factor trials were designed to select the optimal conditions. The effects of different concentrations of PVA (0%, 1%, 2%, 3%, and 4%), SA (1%, 2%, 3%, 4%, and 5%), and DE (0.25%, 0.5%, 1%, 1.5%, and 2%) were assessed. The OD_620_ ranges of *L*. *plantarum* were 0.3, 0.5, 1.0, 1.5, and 2.0. The remaining conditions were performed according to the fermentation method with the above‐mentioned procedures.

#### 2.5.3 Optimization of the DECIMPs fermentation procedure

The rice flour was ground and sifted through an 80‐mesh sieve. The pellets were placed in 50 g rice flour soaked in Milli‐Q water for varying time intervals followed by filtration. Thereafter, rice flour was dried at 40°C after centrifugation and washed three times with Milli‐Q water. The amount of pellets (weight of pellets: weight of rice flour = 1:5, 2:5, 3:5, 4:5, and 5:5), the solid‐liquid ratio (1:1, 1:2, 1:3, 1:4 1:5, 1:6, and 1:7), and the time (12, 24, 36, 48, and 60 hr) were selected to examine the optimal reaction conditions.

#### 2.5.4 Determination of the adsorption property of DECIMPs

1ml of free microorganism (OD_620_ = 1.5) and the DECIMPs containing the same amount of microorganisms were placed in 50‐ml solution of CaCl_2_ (4 μg/ml), respectively. And the mixture was stirred (80 rpm) in a constant temperature shaker at 37°C for 22 hr. A sample of the solution was taken every hour for measurements.

### 2.6 Determination of the physicochemical properties of rice before and after fermentation

Accurately weighted 50.0 g rice was rinsed twice with ultrapure water after fermentation under optimal conditions. The fermented rice was placed into a constant temperature blast drying oven at 40°C for 48 hr. The moisture, protein, fat, and ash contents of rice before and after fermentation were determined according to Chinese national standards (GB/T5497‐85, GB5009.5‐2010, GB/T5512‐2008, and GB5009.4‐2010). Starch content was determined using a total starch content kit. A scanning electron microscopy (SEM, S‐3000N; Hitachi), X‐ray diffraction spectroscopy (XRD, Empyrean, PANalytical) analysis, Fourier‐transform infrared spectroscopy (FTIR, NEXUS670, Thermo Nicolet Corporation), and differential scanning calorimetry (DSC, TA instruments) detection were performed for rice samples before and after microbial fermentation.

### 2.7 Statistical analysis

All statistical analyses were performed in Excel 2010 and SPSS 17.0 statistical analysis software. All experimental data are presented as the mean ± standard deviations (*SD*) of three parallel samples.

## 3 RESULTS AND DISCUSSION

### 3.1 Microbial fermentation for Cd removal

**FIGURE 1:**
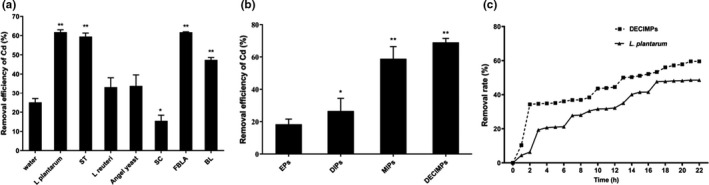
Rate of cadmium removal. (a) Rate of cadmium removal with various stains. (b) Rate of cadmium removal with various pellets. (c) Adsorption curves of *L*. *plantarum* and DECIMPs. ** and * indicate that the difference was extremely significant at *p* values <.01 and <.05

According to Figure 1, the *L*. *plantarum* (61.80%) and *FBLA* (61.71%) on the removal of Cd from rice were significantly enhanced. *L*. *plantarum* has a strong ability to produce acid, which is considered as an important characteristic for Cd removal (Zhai et al., 2019). At the same time, *L*. *plantarum* can be used in fermented foods to product the desirable flavors, inhibits the spoilage microbes and pathogens (Rhee et al., 2011). Therefore, *L*. *plantarum* was selected as the optimal strain.

**FIGURE 2:**
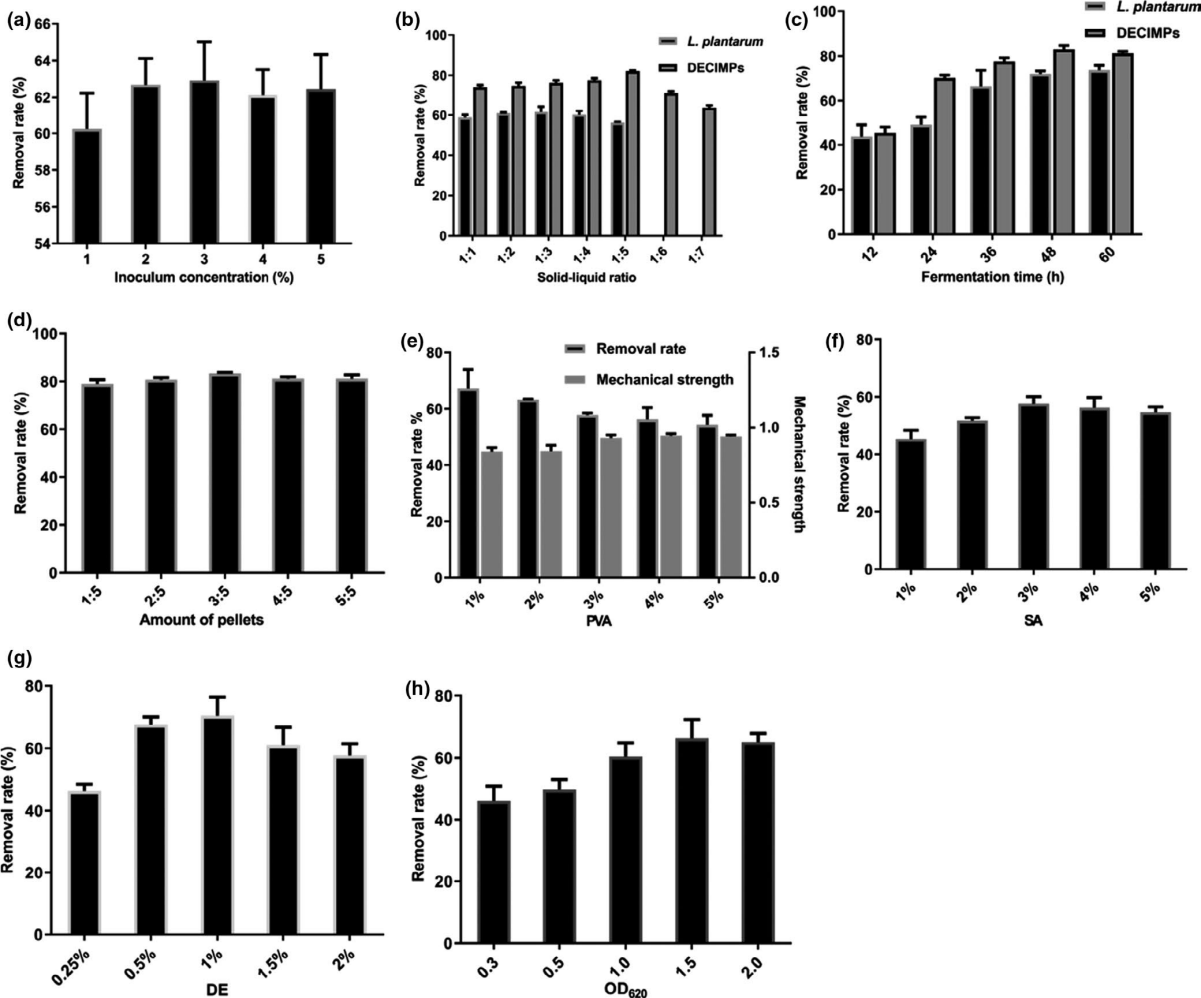
The effects of three factors on the removal rate of cadmium by *L*. *plantarum* and DECIMPs in (a, b, c, and d). Effects of PVA, SA, DE, and OD620 in *L*. *plantarum* suspension on cadmium removal rate by DECIMPs are shown in (e, f, g, and h). Mechanical strength: 60 intact pellets and 100 ml MRS medium were placed into a beaker, use a magnetic stirring device to stirred for 24 hr. The percentage of intact pellets in the total number of original pellets indicates their relative strength. The values are the means ± *SD* of three determinations

As is shown in Figure 2, the optimal fermentation conditions of *L*. *plantarum* are as follows: inoculum concentration (2%), solid‐liquid ratio (1:3), and fermentation time (48 hr). Under these conditions, the Cd removal rate reached 72.03% ± 1.26%, and the concentration of Cd was 0.129 ± 0.005 mg/kg, which is lower than the national limit (0.200 mg/kg) in China (GB2762‐2017).

We examined the optimal fermentation conditions for the inoculum concentration (3.5%), fermentation time (48 hr), and solid‐liquid ratio (1:3) using orthogonal experiments. The removal of Cd (80.02% ± 0.01%) under the optimal conditions predicted by the orthogonal conditions was verified, and it was higher than the optimal condition in single‐factor experiments. The removal of Cd from sample 2 was 77.25% ± 1.05%, and the concentration of Cd decreased to 0.199 ± 0.390 mg/kg.

**FIGURE 3:**
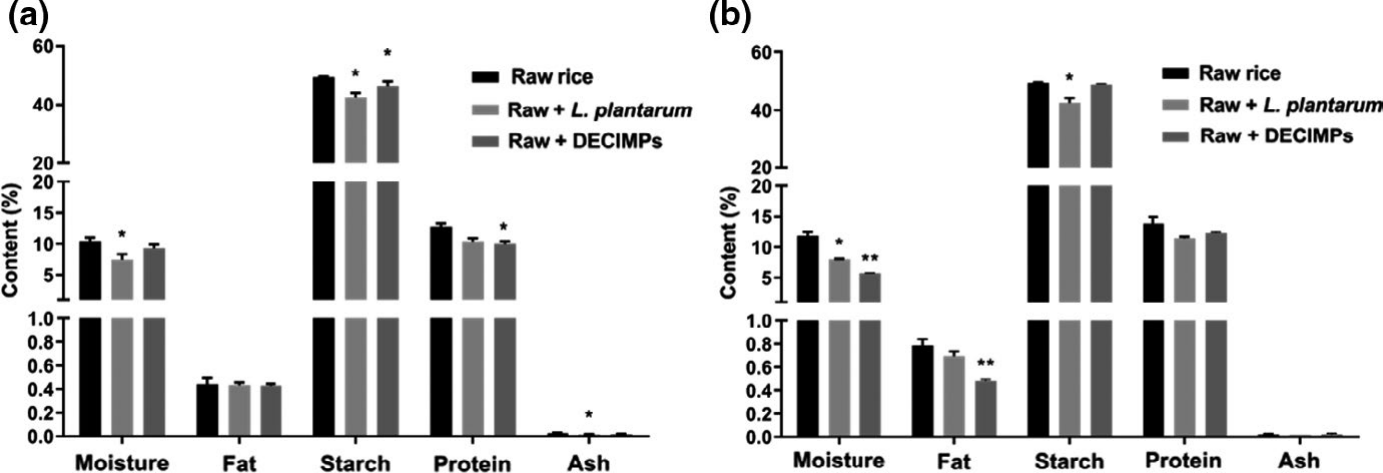
Nutrition of raw rice before and after fermentation. (a and b) are samples 1 and 2 ** and * indicate that the difference was extremely significant at *p* values <.01 and <.05

**FIGURE 4:**
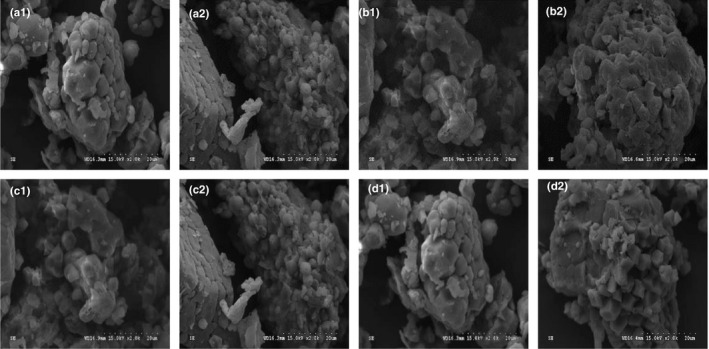
Scanning electron microscopy images of raw rice and fermented rice surface. (a and b) are rice before and after fermentation by *L*. *plantarum*. (c and d) are rice before and after fermentation by DECIMPs. 1 and 2 indicate sample 1 and sample 2

**FIGURE 5:**
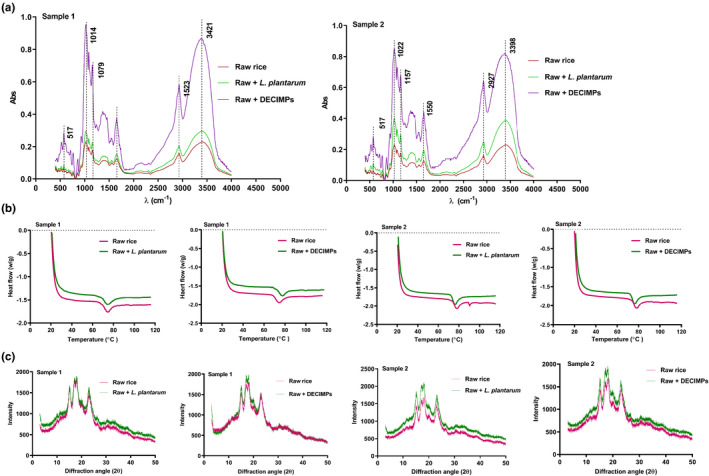
Physicochemical properties of rice before and after fermentation. (a) FTIR of rice flour. (b) DSC gelatinization curves of rice flour. (c) X‐ray diffraction spectra of rice flour

The levels of moisture, protein, and starch contents in two rice samples decreased after fermentation (Figure 3). Cd mainly binds to proteins in rice and forms a stable complex. During microbial fermentation, the proteins in rice were dissolved by acids, which are beneficial to Cd removal (Nguyen et al., 2013; Zhai et al., 2019). Two samples had a loose structure with obvious interstice (Figure 4). The X‐rays show that the crystallinity of sample 2 decreased from 16.23% to 11.41% (Figure 5c). There were no significant variation and a new absorption peak in the FTIR spectra (Figure 5a).

### 3.2 Diatomaceous earth coimmobilized microbial pellets fermentation for Cd removal

The adsorption rate of EPs was 18.40% ± 7.83% (Figure 1b). Compared with the EPs, the adsorption capacity of DIPs for Cd was significantly improved (*p* < .05), and the removal rate of MIPs reached 58.86% ± 7.56%. DECIMPs (69.20% ± 2.48%) were significantly improved compared with other methods. Cd is adsorbed via a complexation reaction, ion exchange, physical adsorption, and intracellular diffusion (Chakravarty & Banerjee, 2012). Cell components of *L*. *plantarum*, such as the hydroxyl group and phosphate group, are involved in the adsorption of Cd (Gerbino et al., 2011). The infiltration of DE makes the gel loose and porous, so DECIMPs have better adsorption properties (Xiong & Peng, 2008).

As is shown in Figure 2e, the gel strength of the pellets was enhanced with the increase in PVA, but the adsorption rate decreased gradually. PVA, as a cross‐linking agent to make the cross‐linked porous structure too dense, the pores become smaller, and the surface area decreases (Yañez‐Ocampo et al., 2009). The Cd removal rate varied with SA content and decreased to 3%. When the DE content was 1% (Figure 2g), the removal rate of Cd reached its highest. Therefore, SA (3%), DE (1%), PVA (2%), and OD_620_ (1.5) should be used as the optimization conditions in future studies.

As shown in Figure 1c, the adsorption rate of immobilized microorganisms was always higher than nonimmobilized microorganisms. Because the carrier provides nutritional for the microorganism, the thick gel layer hinders the direct contact of substrates, pollutants, and degradation products with microorganisms, and the addition of adsorbent in the carrier is conducive to the transfer of substances in the carrier (Wang et al., 2012); the microorganism fixed on the carrier may be more protected from CaCl_2_ solution (Tarabukin et al., 2017), which increased the activity of microorganisms and improved the adsorption capacity of Cd.

Under optimal conditions (Figure 2), amount of pellets (3:5, immobilized microbial mass: rice flour mass), solid‐liquid ratio (1:5), and fermentation time (48 hr), the removal rate reached 83.62% ± 2.20%, and the Cd content in rice was 0.083 ± 0.010 mg/kg, which is lower than the national limit in China.

The order of Cd removal by various influencing factors (amount of pellets>exposure time>solid‐liquid ratio) was identified using the orthogonal test. The optimal conditions were predicted as follows: amount of pellets (3.5:5), fermentation time (54 hr), and solid‐liquid ratio (1:5.5). The Cd removal rate reached 87.13% ± 0.05%, which was lower than the no. 3 experiment in the orthogonal experiment (the data were not shown). We performed the experiment again under the following conditions: amount of pellets (2.5:5), fermentation time (54 hr), and solid‐liquid ratio (1:5.5). The Cd removal rates reached 90.01% ± 1.01% (sample 1) and 91.80% ± 0.54% (sample 2). The Cd contents in samples 1 and 2 were 0.051 ± 0.003 mg/kg and 0.068 ± 0.034 mg/kg, which are lower than the national limit in China.

The fat content of sample 2 and the proteins in sample 1 was significantly reduced (Figure 3). The pore size and structure of the two kinds of rice became larger and looser, after immobilized microbial fermentation compared with the unfermented rice flour (Figure 4), but the changes were not as obvious as the direct microbial fermentation.

As can be seen in Figure 5b, the gelatinization temperature of the sample 1 and sample 2 were decreased, the gelatinization time was prolonged. The crystallinity of the two kinds of rice flour was decreased to different degrees (Figure 5c). Fermentation destroys the double helix structure of amylopectin and forms hydrogen bonds with acids, which decreases crystallinity (Li et al., 2019). The structure of rice flour was determined using FIRT (Figure 5a). The shape of absorption peaks (O‐H, C‐H, C=O, C‐H, and C‐H) did not change, and the intensity of absorption peaks increased slightly, these indicate no obvious chemical change in the rice flour during the fermentation process of immobilized microorganisms, and no new chemical bonds or groups formed.

## 4 CONCLUSION

In conclusion, this study confirmed that DECIMPs fermentation can markedly reduce Cd levels in rice, the Cd removal rates reached 90.01% ± 1.01% (sample 1) and 91.80% ± 0.54% (sample 2). In addition, the effect on rice quality was smaller, microbial was easily separated. These advantages made up for the disadvantage that traditional microorganisms cannot be used to remove Cd from powdery materials and provide a new method for the repair of food contaminated by heavy metals.

## CONFLICT OF INTEREST

The authors declare that they have no conflict of interest.

## AUTHOR CONTRIBUTIONS

**Fang Zhao:** Data curation (lead); Investigation (equal); Methodology (equal); Writing‐original draft (lead). **Hu Zhang:** Data curation (equal); Investigation (equal); Methodology (equal); Writing‐original draft (equal). **Pianpian Yan:** Conceptualization (equal); Methodology (equal); Resources (equal); Visualization (equal). **Yuwei Chen:** Investigation (equal); Methodology (equal); Software (equal). **Qian Wu:** Investigation (equal); Methodology (equal); Visualization (equal). **Min Fang:** Resources (equal); Validation (equal); Visualization (equal); Writing‐review & editing (equal). **Yongning Wu:** Supervision (lead); Visualization (equal). **Zhiyong Gong:** Supervision (equal); Visualization (equal); Writing‐review & editing (equal).

## ETHICAL APPROVAL

This article does not contain any studies with human participants or animals performed by any of the authors.
